# Characterisation of the Phenolic Profile of *Acacia retinodes* and *Acacia mearnsii* Flowers’ Extracts

**DOI:** 10.3390/plants11111442

**Published:** 2022-05-28

**Authors:** Soraia I. Pedro, Tiago Rosado, Celina Barroca, Duarte Neiva, Vanesa Alonso-Herranz, Ana Gradillas, Antonia García, Jorge Gominho, Eugenia Gallardo, Ofélia Anjos

**Affiliations:** 1 Centro de Biotecnologia de Plantas da Beira Interior, 6001-909 Castelo Branco, Portugal; soraia_p1@hotmail.com (S.I.P.); celinasbarroca@gmail.com (C.B.); 2 Centro de Estudos Florestais (CEF), Instituto Superior de Agronomia, Universidade de Lisboa, 1349-017 Lisboa, Portugal; duarteneiva@isa.ulisboa.pt (D.N.); jgominho@isa.ulisboa.pt (J.G.); 3 Centro de Investigação em Ciências da Saúde (CICS-UBI), Universidade da Beira Interior, 6200-506 Covilhã, Portugal; tiago.rosado@ubi.pt (T.R.); egallardo@fcsaude.ubi.pt (E.G.); 4 Laboratório de Fármaco-Toxicologia—UBIMedical, Universidade da Beira Interior, 6200-506 Covilhã, Portugal; 5 Instituto Politécnico de Castelo Branco, 6001-909 Castelo Branco, Portugal; gradini@ceu.es (A.G.); antogar@ceu.es (A.G.); 6 Centro Ecologia Aplicada “Prof. Baeta Neves” (CEABN), Instituto Superior de Agronomia, Universidade de Lisboa, 1349-017 Lisboa, Portugal; 7 (CEMBIO) Centro de Metabolómica y Bioanálisis, Facultad de Farmacia, Universidad San Pablo CEU, CEU Universities, Campus Monteprincipe, Boadilla del Monte, 28660 Madrid, Spain; valonso@ceu.es

**Keywords:** *Acacia* species, flowers, phenolic compounds, flavonoid content, UHPLC/ESI-QTOF-MS, HPLC-DAD, FTIR-ATR

## Abstract

*Acacia* spp. is an invasive species that is widespread throughout the Portuguese territory. Thus, it is pertinent to better understand this species in order to find different applications that will value its use. To evaluate the phenolic profile in Acacia flowers, ethanolic extracts obtained through an energized guided dispersive extraction were analysed, focusing on two species, *Acacia retinodes* and *Acacia mearnsii*, at two flowering stages. The phytochemical profile of each extract was determined by ultra-high performance liquid chromatography coupled with quadrupole time-of-flight mass spectrometry and high-performance liquid chromatography coupled with diode array detector. The FTIR-ATR technique was used to distinguish the different samples’ compositions. The results showed the presence of high concentrations of phenolic compounds (>300 mg GAE/g extract), among which are flavonoids (>136 mg QE/g extract), for all combinations of species/flowering stages. The phytochemical profile showed a complex composition with 21 compounds identified and quantified (the predominant ones being epicatechin, rutin, vanillin, and catechol). Both species and flowering stages presented significant variations regarding the presence and quantity of phenols and flavonoids, so much so that a principal component analysis performed with FTIR-ATR spectra data of the extracts was able to discriminate between species and flowering stages.

## 1. Introduction

Natural compounds obtained from plants offer research opportunities due to their significant pharmacological and toxicological properties [1]. They are often considered as potential new drugs against drug-resistant pathogens [2] and for treating diseases.

A variety of medicinal species belonging to the genus *Acacia* suggest the potential presence of bioactive metabolites. The most characteristic in this genus are flavonoids and tannins compounds [3]. The literature that is focused on plant extracts indicates that these species are rich in phytochemical compounds such as polyphenols, flavonoids, saponins, alkaloids, among others. The accumulations are made in different tissues/organs (leaves, barks, flowers, seeds, wood, and twigs) and have wide applications as antioxidants, antimicrobials, pharmaceuticals, and biopesticides [4,5,6,7,8].

Acacia flowers play a crucial role in colonisation ability. They limit the growth of other species due to allelopathic interference caused by the decomposition of this plant material in the soil, altering its characteristics to decrease other species’ viability and, therefore, increasing its unopposed proliferation. This effect is longstanding as the soil toxicity percolates for a long time [9,10]. Flower extracts are already exploited in hydrogels for personal care products, cosmetics, pharmaceuticals, and perfumes based on their anti-radical and anti-proliferative potential [11].

The species selected for this study are *Acacia mearnsii* De Wild. and *Acacia retinodes* Schltdl., belonging to the Fabaceae Lindl. family, Caesalpinioideae subfamily, Acacieae tribe, and *Acacia* genus [12,13,14,15]. *A. mearnsii* is commonly known as “Acacia-Negra” or Black Wattle [13], a native from southeastern Australia and introduced in Africa, the Caribbean, east Asia, Europe, Sri Lanka, North America, New Zealand, South America, and southeast Asia [16,17]. It is considered a rapidly growing tree species of economic importance in Japan as well as an important commercial forestry species in South Africa, mainly due to the abundance of tannin in its bark and for pulp in the pulp and paper industry [11]. Traditionally, its bark extract has been used in the leather tanning industry. Moreover, current research on the composition and biological activity of the bark extract has suggested applications in health and medicine [18].

*A. retinodes* is endemic to south Australia, yet has been introduced to Africa, Europe, Macronesia, North America, the Pacific and Indian Ocean Islands, the Indian subcontinent, and southeast to west Asia [16,17], known as Wirilda, Swamp Wattle, or Silver Wattle [13]. According to O’Leary (2007) [19], *A. retinodes* bark was mentioned, in 1848, for its use in tanning, with its exuded gums becoming an early export commercial business and with natives eating it due to its nutritious characteristics. Acacia barks have been the focus of some studies regarding their extractives’ contents and bioactive components [11,20], with a significant fraction being attributed to tannins and related compounds. On the other hand, little work has been conducted on Acacia. Correia et al. [11] reported that *Acacia* spp. flowers are economically important due to their application in the cosmetic and perfume industries. However, there is a lack of information regarding the flower’s potential, as raw material, to derive renewable phenolic compounds from such a widely distributed species. More research on these materials can complement other end-uses, already associated with different plant parts of these species, intending on total biomass utilisation in a zero-waste philosophy.

New emerging technologies used for the extraction of bioactive compounds are gaining attention at both industrial and scientific research scales [21,22] when compared to conventional extraction procedures such as solid–liquid extraction [23] or Soxhlet extraction [24]. These new technologies can save time, energy, and specific resources, reducing the carbon footprint in extraction processes [24]. In this context, Energized Dispersive Guided Extraction (EDGE), an automated extraction system, can be considered an innovative procedure. This simple and efficient method translates into high extract yields by combining heat, pressure, and solvents to quickly and efficiently extract samples. This methodology has been used to extract Oregon List pesticides from cannabis edibles (candy, French fries, chocolate, cookies, and granola) and cannabis flowers and to compare the efficiency of extraction of phenolic compounds present in Acacia flowers using EDGE procedure and the Soxhlet apparatus (classical method) [25]. The extraction by EDGE showed a more efficient extraction and a faster, cleaner, and safer controlled methodology, which is in line with the time and resource savings and increased extraction yields compared to other conventional techniques. One of the novelties of this work lies in the extraction method for the recovery of phenolic compounds and flavonoids from flowers.

Phenolic compounds are the most promising secondary metabolites found in plants due to their potential as natural antioxidants that can be used in the food and pharmaceutical industries.

The present study aims to evaluate the phenolic compounds present on ethanolic extracts of *A. mearnsii* and *A. retinodes* flowers in two flowering stages, using high-performance liquid chromatography (HPLC) coupled to a diode array detector (DAD) and ultra-high-pressure liquid chromatography accurate-mass quadrupole time-of-flight mass spectrometry with electrospray ionization (UHPLC/ESI-QTOF-MS). Fourier-transform infrared spectroscopy—attenuated total reflection (FTIR-ATR) was used to distinguish between species and flowering stages.

Despite all ethnobotanical knowledge about this species, little is known about these species’ chemistry and bioactive compounds, namely *A. mearnsii*, due to the difficulty of *Acacia* species identification apart from their taxonomic relationship [26]. Therefore, the data gathered with this study will allow further research and potential application of these species’ flowers regarding value-added products.

## 2. Results and Discussion

### 2.1. Determination of Total Phenolic and Flavonoids Content

Phenolic compounds are among the main secondary metabolites of plants, and it is possible to find some of them in all plants [27]. These compounds have beneficial properties in the food industry and have medicinal applications as preservatives, antioxidants, anti-inflammatories, and anti-carcinogens, among others [28,29,30]. Total phenolics were determined using the Folin–Ciocalteu colorimetric method (Table 1).

The amount of TPC in flowers from the two flowering stages ranged, on average, between 300 and 350 mg GAE/mL for both *A. mearnsii* and *A. retinodes*.

The ANOVA results (Table 1) show a difference in total phenolic contents between the two species, where this difference accounted for 49.1% of the total variance for TPC. While, for *A. retinodes*, phenolic content increases as the flowering stage progresses, the opposite was observed for *A. mearnsii,* although with a less significant variation. In the species A. *retinodes*, the total phenolic content is higher in the late flowering stage (LF). However, in both species, no significant differences were observed between flowering stage. Flavonoids have beneficial biological activities, namely anti-inflammatory, antimicrobial, antioxidant, cytotoxic, and antitumour [31]. In this study, flavonoids were determined by the aluminium chloride colorimetric method (Table 1).

Concerning TFC, the flowers of the *A. mearnsii* species presented a significantly higher value in relation to the other species analysed, and this difference represented 46.8% of the total variation observed. In this case, the state of maturity was also highly significant (25.9% of the total variance), with the late flowers possessing a higher amount of TFC, independently of species.

These results are similar to those obtained by *A. dealbata* [31], but in this case, the early flowering stage presents higher TPC and TFC than the late stage.

The TPC and TFC obtained in this study are in agreement with those observed for *A. podalyriifolia* flowers [32] but are lower than those observed for *A. confusa* [33].

### 2.2. Targeted and Untargeted Phytochemical Study

The samples were analysed by ultra-high performance liquid chromatography (UHPLC) coupled to a QTOF-MS detector in order to identify the compounds and complement phytochemical characterization. The chromatographic analysis was performed using optimized conditions, providing a satisfactory separation in less than 25 min [34].

Analysis of phenolic compounds is reported in positive and negative ionization modes, but the latter mode was found more sensitive for the analysis of most compounds.

(i) Targeted analysis: Twenty-nine compounds were unambiguously identified and characterised by comparison of retention times and accurate mass spectra with those of authentic standards. A phytochemical library of 48 standard solutions (freshly prepared) was used to identify the metabolites in methanol extracts [35], and two groups of compounds were mainly present: hydroxybenzoic acids and flavonoids (Table 2).

The samples were then analysed by a high-performance liquid chromatography-diode array detector (HPLC/DAD) to identify the other compounds and further complement the initial detection and phytochemical characterisation. Our group has some experience concerning the quantification of phenolic compounds [36,37,38,39]. Thus, compound identification was carried out by comparing their retention times with those obtained from analytical standards. The concentration of the identified compounds was estimated by comparing their peak areas in the chromatograms of plant extracts with calibration curves constructed using the corresponding standard solutions (Supplementary Material: Table S1. Data validation). The results are shown in Table 3. It was in the species *A. retinodes* that it was possible to quantify most of the compounds, 20 of them, while in the species *A. mearnsii,* it was only possible to quantify gallic acid, vanillin, syringaldehyde, caffeic acid, *p*-coumaric acid, trans-cinnamic acid, 5-methylfurfural, (+) catechin, quercetin, and 4′,5,7-trihydroxyflavanone. The remaining compounds were not detected or were below the limit of quantification.

The results presented in Table 4 report the effects of species and flowering stages for all analysed compounds. The percentage of the variance of each factor analysed (species and flowering stages) was calculated based on the two-way ANOVA results.

Regarding Table 4, the concentration of gallic acid, syringaldehyde, and caffeic acid is not affected by species or flowering stages. Vanillin, *p*-coumaric acid, trans-cinnamic acid, 4′,5,7-trihydroxyflavanone, 5-methyfurfural, quercetin, 4′,5,7-trihydroxyflavanone, catechol, and (-)-epicatechin are highly influenced by the species. Comparing the compounds that appear in both species, it is possible to conclude that *A. mearnsii* is richer in the analysed compounds, with a significantly higher amount of vanillin, *p*-coumaric acid, *trans*-cinnamic acid, 4′,5,7-trihydroxyflavanone, catechol, and (-)-epicatechin, where quercetin and 5-methyfurfural are present in a significantly higher amount in *A. retinodes* (Table 3). Gallic acid, syringaldehyde, and caffeic acid do not show significant differences between species.

Furfural, 4-hydroxybenzoic acid, chlorogenic acid, 4-hydroxybenzaldehyde, myricitrin, rutin, kaempferol, and myricetin are presented only in the *A. retinodes* species (Table 3), and the differences between early flower and late flower are highly significant (Table 4). Furfural, 4-hydroxybenzoic acid, myricitrin, myricetin, and rutin present as significantly higher on LF than in EF.

Only chlorogenic acid and kaempferol are present in a significantly higher amount in EF.

The compounds (+)-catechin and coniferaldehyde appear only in *A. mearnsii* but do not show significant differences between LF and EF (Table 3 and Table 4).

For the remaining compounds whose quantification was not performed by HPLC-DAD due to the absence of analytical standards, the concentration of the identified compounds by UHPLC/ESI-QTOF-MS was estimated by comparing their peak areas in the chromatograms from the plant extracts with those of the corresponding standard solutions freshly prepared and analysed by duplicate in the same batch as the samples (Supplementary Material Table S2). These results are presented in Table 5.

(ii) Untargeted study: Finally, UHPLC-ESI-QTOF-MS/MS facilitates the characterization of known and unknown compounds on the basis of their molecular formula, exact mass measurements, and MS/MS fragmentations. Thus, the most abundant signal was analysed in both species (Supplementary Material: Figures S1 and S2). The compounds were tentatively identified by matching the accurate masses (±5 ppm error) of the detected molecular ions and their MS/MS patterns (Table 6). These patterns were obtained from those compounds previously reported in the literature and after comparison against open-acess databases such as FOODB (http://foodb.ca, accessed on 2 February 2022) [40] and by using the on-line tool CEU Mass Mediator (http://ceumass.eps.uspceu.es/mediator, accessed on 2 February 2022) [41].

A global analysis was performed using a heat map to visualise hierarchical clustering that ordered similar groups to understand the behaviour of the different analysed compounds as a whole concerning each species and in the different flowering states to understand coherent patterns among them.

A heat map is a graphical representation of data where the individual values contained in a matrix are represented as colours [42].

**Figure 1:**
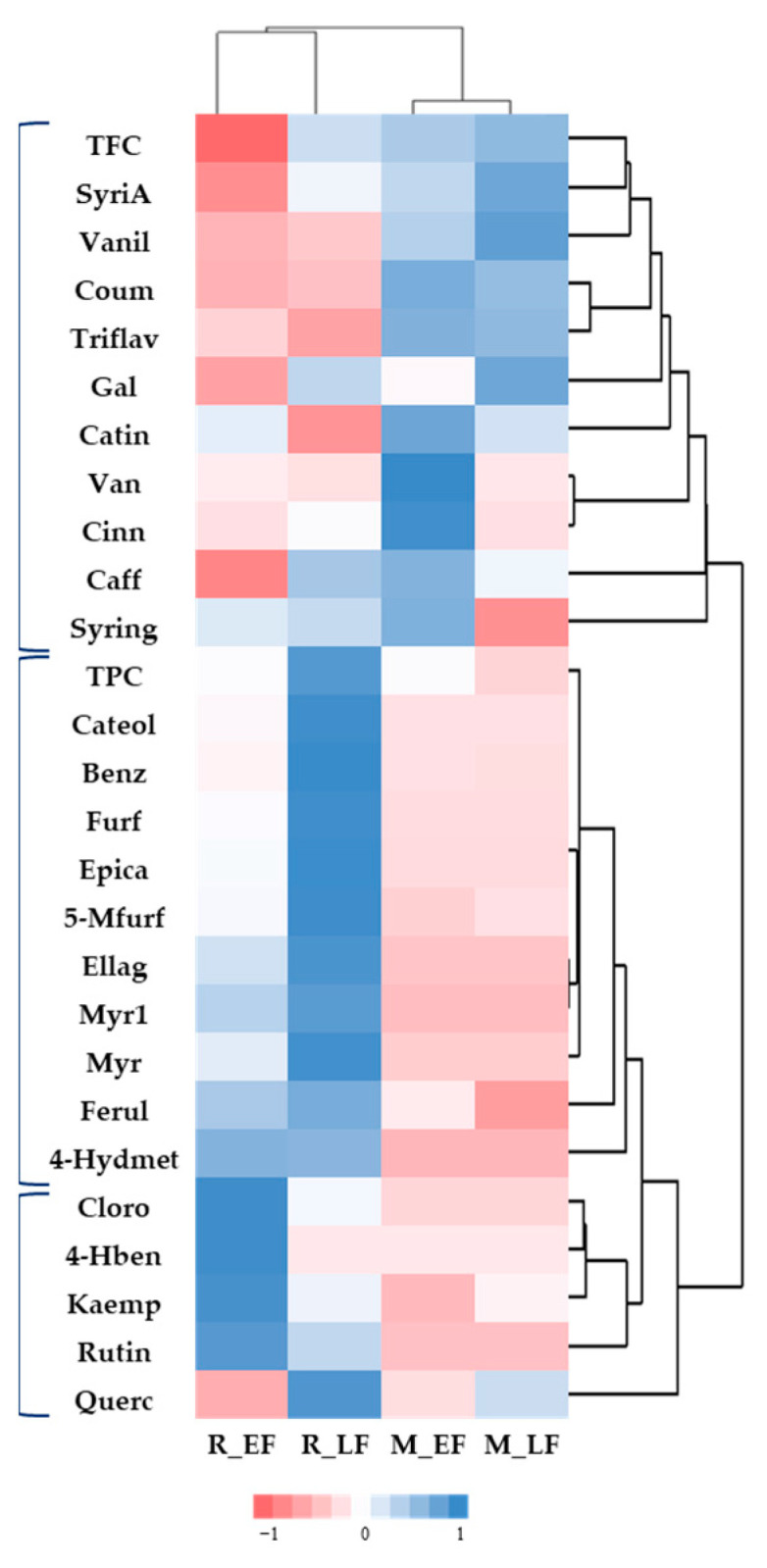
Heat maps plotting clusters of contents of phenol compounds, TPC and TFC analysed by HPLC of the *A. retinodes* and *A. mearnsii* flowers with different flowering stages. TFC—Total phenolic compounds; Vanil—Vanillin; Coum—*p*-Coumaric acid; Triflav—4′,5,7-Trihydroxyflavanone; Gal—Gallic acid; Catin—(+)-Catechin; Cinn—trans-Cinnamic acid; Caff—Caffeic acid; Syring—Syringaldehyde; TPC—Total flavonoid content; Catol—Catechol; Benz—Hydroxybenzoic acid; Furf—Furfural; Epica—(-)-Epicatechin; 5-Mfurf—5-Methylfurfural; Myrci—Myricitrin; Myr—Myricetin; Conif—coniferaldehyde; Cloro—Chlorogenic; 4-Hben—4-Hydroxybenzaldehyde; Kaemp—Kaempherol; Rutin—Rutin; Querc—Quercetin; R_EF—*A. retinodes* early flower; R_LF—*A. retinodes* late flower; M_EF—*A. mearnsii* early flower; M_LF—*A. mearnsii* late flower.

The heat map (Figure 1) was generated from the content of compounds identified in each species and flowering stage. The blue colours represent a positive correlation between analyte levels and species, while the pink colours depict a negative correlation.

Heat maps clustered the two *Acacia* species into two groups according to the abundance and presence of the different compounds. Total phenolic compounds, gallic acid, caffeic acid, (+)-catechin, *p*-coumaric acid, 4′,5,7-trihydroxyflavanone, trans-cinnamic acid, vanillin, and syringaldehyde are more abundant in *A. mearnsii*, with some differences between EL and LF. *A. retinodes* presents a higher amount of the other compounds. Some of them appear only in this species, as aforementioned.

Regarding the cluster where *A. retinodes* have a higher amount, it was also possible to identify two sub-clusters related to the differences between early flowers and late ones.

### 2.3. FTIR-ATR Spectral Analysis

**Figure 2:**
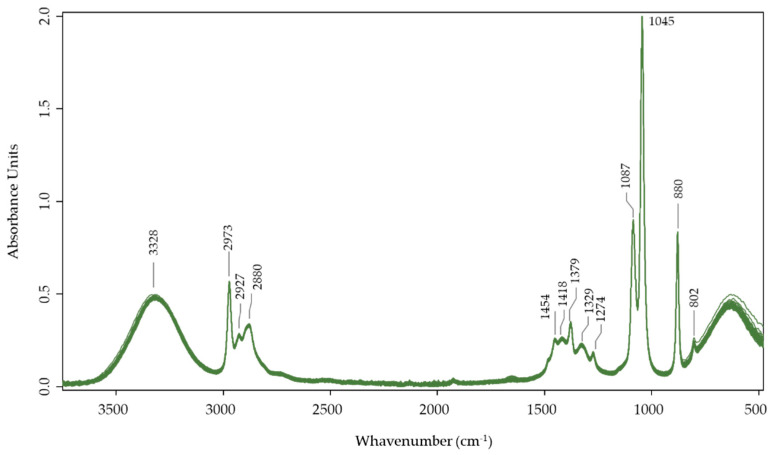
FTIR-ATR spectrum for all Acacia flower samples.

The spectra obtained with the ethanolic flowers’ extract (Figure 2) display the strong influence of the compounds in this matrix. These results are similar to those obtained for rosemary [43], Cucurbitaceae [44], and medicinal plant extracts [45].

The intense band at 3328 cm^−1^ was assigned by the stretching vibrations of -OH groups more highly influenced by water content in the samples. Still, a lower strength could also affect some alcohols, phenols, or peroxides.

The bands at 2973 cm^−1^, 2927 cm^−1^, and 2880 cm^−1^ are characteristics of C-H stretching vibrations mainly to -CH_3_ and -CH_2_ as well as aromatic groups present in some plant extracts and the ethanol present in the matrix. These peaks can also be related to the diterpene three-ring structure [44].

The band at 1454 cm^−1^, corresponding to the combination of bending vibration of -CH_2_ and the vibration of the COO– group in the flavanol and organic acids [46] and also related to -CH_3_ in acetyl groups and at 1418 cm^−1^, can be assigned by -CH_2_ and -OCH_3_ groups. The peak found at 1379 cm^−1,^ and 1329 cm^−1^ can be related to C-H’s symmetric deformation vibrations in methoxy groups and phenolic hydroxyls [47].

The peak at 1274 cm^−1^ could be related to asymmetric stretching vibrations of the C-O-C linkages in phenolic ethers (C-O stretch) and esters of phenolic hydroxyls [43].

The strong bands at 1087 cm^−1^ and 1045 cm^−1^ are related to the primary alcohol, C-O stretch, and the secondary alcohol [48].

At 880 and 802 cm^−1^, it is possible to find the peaks associated with the C-C skeletal vibration and out-of-plane bending vibrations associated with some aromatic ring (aryl) group frequencies [48].

**Figure 3:**
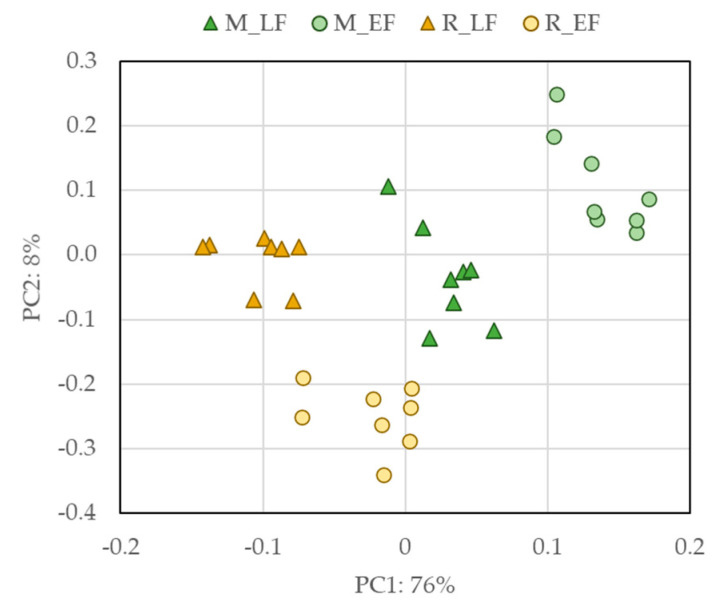
Score plot of the first two principal components of the PCA performed with FTIR-ATR spectra of Acacia flower samples using the first derivative Savitzky–Golay spectra transform with 17 smoothing points. M_LF—*A. mearnsii* late flower; M_EF—*A. mearnsii* early flower; R_LF—A. *retinodes* late flower; R_EF—A. r*etinodes* early flower.

Figure 3 shows the PCA plot of the FTIR-ATR spectra obtained with all the samples. This non-supervised multivariate statistical analysis reveals that this technique is able to discriminate between flower raw materials based on their differences, among which their phenolic and flavonoid profile of the Acacia’s species and flowering stages have their impact. Similar results were found, but with FT-RAMAN, in a previous work [49]. The two first components can justify 84% of the variation being the first component capable of distinguishing between the two species.

## 3. Materials and Methods

### 3.1. Plant Material

**Figure 4:**
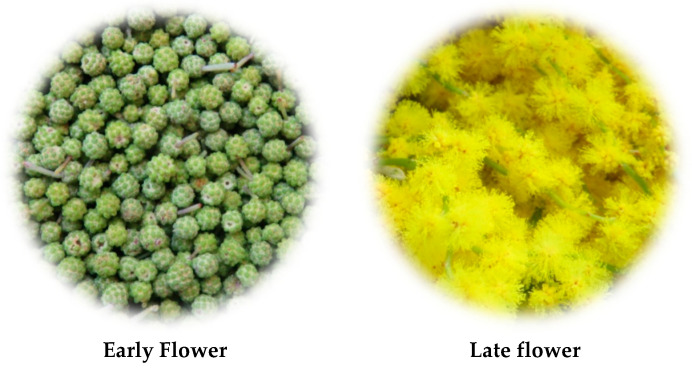
Examples of different flowering stages used in this study.

The flowers of *A. retinodes* and *A. mearnsii* were harvested during two flowering stages (early flower—EF; and late flower—LF). They were collected in March 2021 in the Lisbon region (*A. retinodes* at 38.747067; −9.274577 and *A. mearnsii* at 38.713910, −9.192827). The flowers were freeze-dried and kept at −80 °C until extraction. Figure 4 shows an example of the different flowering stages.

### 3.2. Chemicals and Reagents

Purified water was obtained using the Milli-Qplus185 system (Millipore, Billerica, MA, USA). LC–MS grade solvents and formic acid (MS quality) from Fisher Chemical were used (Waltham, MA, USA). Standards of chlorogenic acid (5-*O*-caffeoylquinic acid), epicatechin, kaempferol, myricitrin, myricetin, cyanidin-3-*O*-glucoside, and quercetin-3-*O*-glucoside were obtained from Extrasynthesis Phytochemicals (Genay Cedex, France), and catechol and 4-Hydroxybenzoic acid were obtained from Alfa Aesar (Kandel, Germany). The remaining standards (all >99.5%) and reagents were obtained from Sigma-Aldrich (St. Louis, MO, USA). Standard stock solutions were prepared in methanol (10 mg/L) and also diluted with methanol to obtain working standard solutions.

### 3.3. Extraction Conditions

According to the procedure previously described, the energized dispersive guided extraction (EDGE) equipment was used for extraction [25].

The flower samples were ground in a hammer mill with a 1 mm mesh in the first step. After that, 1 g of powder sample underwent three successive extraction cycles using 20 mL of ethanol for 10 min at 40 °C in each cycle. The crude material of each sample was weighed directly into a Q-Cup containing a sandwich of tree Q-Discs composed of one fibreglass filter surrounded by two cellulose filters.

All extractions were performed in duplicate, and all subsequent measurements and analyses were performed in triplicate.

### 3.4. Determination of Total Phenolic and Flavonoid Contents

Total phenolic content (TPC) was determined by the Folin–Ciocalteu colorimetric method [50], using gallic acid as a standard. The ethanolic solution of each extract or standard (50 µL) was mixed with 450 µL of distilled water. Then, 2.5 mL of Folin–Ciocalteu reagent (0.2 N) was added, leaving the mixtures for 5 min before adding 2 mL of aqueous Na_2_CO_3_ (75 g/L). The reaction mixtures were incubated for 90 min at 30 °C. Total phenols were determined by colourimetry at 765 nm.

The calibration curve was prepared using gallic acid standard solutions with concentrations between 0.016 and 3.2 mg/L (y = 0.2249 x; R^2^ = 0.9973). TPC was expressed as gallic acid equivalents (mg GAE/g extract).

The total flavonoids content (TFC) was determined by the aluminium chloride colorimetric method according to Luís et al. [51].

Each ethanolic solution of the extracts (500 μL) was mixed with methanol (1.5 mL), 10% aluminium chloride (0.1 mL), 1 M potassium acetate (0.1 mL), and distilled water (2.8 mL). This solution was left in the dark at room temperature for 30 min, and the absorbance of the reaction mixture was measured at 415 nm using a spectrophotometer. The calibration curve was performed by preparing eight quercetin solutions at concentrations between 2.5 and 12.5 mg/L in methanol (y = 0.0557 x; R^2^ = 0.9925). TFC was expressed as quercetin equivalents (mg QE/g extract).

### 3.5. UHPLC/ESI-QTOF-MS Analysis

The secondary metabolites present in the flower samples were identified using a methodology previously developed at the Center of Metabolomics and Bioanalysis (CEMBIO) by UHPLC/ESI-QTOF-MS [34]. Briefly, 500 μL of methanol was added to an extract obtained from lyophilized flowers (1 g). The mixture was vortexed for 2 min, sonicated for 15 min, and centrifuged at 10,000× *g* for 10 min at 4 °C. The supernatant was transferred to a Chromacol vial (Thermo Fisher Scientific, Madrid, Spain) for LC/MS analysis. The procedure was performed in duplicate. The samples were analysed on a 1290 Infinity series UHPLC system equipped with an electrospray ionization source (ESI) with Jet Stream technology coupled to a 6545 iFunnel QTOF/MS system (Agilent Technologies, Waldbronn, Germany).

The separation was performed in a RP column Zorbax Eclipse XDB-C_18_ (4.6 × 50 mm, 1.8 µm) (Agilent Technologies) maintained at 40 °C. The flow rate was 0.5 mL/min with a mobile phase consisting of solvent A: 0.1% formic acid in water and solvent B: methanol. The elution was carried out in gradient mode and included 2% B (0–6 min), 2–50% B (6–10 min), 50–95% B (11–18 min), and 95% B for 2 min (18–20 min) and was returned to starting conditions 2% B in one minute (20–21 min) to finally keep the re-equilibration, with a total analysis time of 25 min. The injection volume was 2 μL. The detector was operated in full scan mode (*m/z* 50 to 1500) at a scan rate of 1 scan/s in positive and negative ESI mode. MS/MS information was acquired in the automatic mode (Auto MS-MS scan mode at 30 eV of collision energy).

Accurate mass measurement was assured through an automated calibrator delivery system that continuously introduced a reference solution containing masses of *m/z* 121.0509 (protonated purine) and *m/z* 922.0098 (protonated HP-921) in positive ESI mode, whereas *m/z* 119.0363 (abstracted proton purine) and *m/z* 966.0007 (formate adduct of HP-921) were in negative ESI mode. The capillary voltage was ± 4000 V for negative and positive ionization mode. The source temperature was 225 °C. The nebulizer and gas flow rate were 35 psig and 11 L/min, respectively, with a fragmentor voltage to 75 V and a radiofrequency voltage in the octopole (OCT RF Vpp) of 750 V.

The MassHunter Workstation Software LC/MS Data Acquisition Version B.07.00 (Agilent Technologies) was used for control and data acquisition. LC-QTOF-MS data processing was performed in MassHunter Qualitative Analysis (Agilent Technologies) Software version B.08.00.

### 3.6. HPLC Analysis

A high-performance liquid chromatography (HPLC) system with a binary pump coupled to a diode array detector (DAD) from Agilent Technologies (Soquimica, Lisboa, Portugal) was used to analyse the phenolic compounds. The dried samples were dissolved in 500 µL of absolute ethanol and then filtered (0.22 µm) before being transferred to the autosampler for injection into the HPLC-DAD system.

Compounds except rutin and myricitrin were separated with a YMC-Triart PFP column (5 μm, 4.6 × 150 mm i.d.), with pre-column maintained at 35 °C. A gradient mode phase system consisting of eluent A: acetonitrile and eluent B: 0.1% trifluoroacetic acid in water was used. Separation was achieved with a linear gradient program as follows: 10% A (0–3 min), 10–15% A (3–15 min), 15% A (15–20 min), 15–18% A (20–25 min), 18–30% A (25–40 min), 30–50% A (40–45 min), 50–100% A (45–50 min), after which it returned to the initial conditions, 10% A (50–55 min). The flow rate was 1 mL/min^−1^. The temperature of the sampler was set at 4 °C, and the injection volume was 50 µL. The analytes were detected between 255 and 360 nm.

Concerning the separation of rutin and myricitrin, the mobile phase was composed of (A) 0% acetonitrile and (B) 100% orthophosphoric acid. Separation was achieved with a linear gradient program, as follows: 0% A (0 to 2 min), 9% A (2 to 14 min), 13% A (14 to 22 min), 33% A (22 to 38 min), 43% A (38 to 44 min), kept to 43% A (44 to 55 min), after which it returned to the initial conditions, 0% A (55 to 65 min). The flow rate was 0.8 mL/min^−1^. The temperature of the column and sampler were set at 24 °C and 4 °C, respectively, and the injection volume was 50 µL. The analytes were detected between 255 and 360 nm.

### 3.7. FTIR-ATR Spectral Analysis

Spectra were acquired by the FTIR-ATR (Fourier transform infrared spectroscopic) method with a Bruker spectrometer (Alpha, Bruker Optics GmbH, Ettlingen, Germany) using a diamond crystal. Four spectra per sample were obtained with 16 scans per spectrum, with a spectral resolution of 4 cm^−1^ in the range 4000 to 450 cm^−1^. The background was performed after scanning a sequence of eight samples.

### 3.8. Statistical Analysis

An analysis of variance (ANOVA) was conducted to estimate how the mean of the quantitative variable changes according to the levels of the categorical variables. Based on the results of the ANOVA, an estimate of the percentage of variances of each was made. The LSD test was applied to determine whether individual means differed. A heat map was also performed, representing values for the main variable of interest across two axis variables as a cluster effect. For ANOVA and heat map software, STATISTICA 7 (StatSoft. Inc. United States, USA) was used; for spectral data analysis and principal component analysis (PCA) OPUS^®^, version: 7.5.18 (Bruker Optics, Ettlingen, Germany) and UnscramblerX 10.5 (CAMO, Oslo, Norway) were used.

## 4. Conclusions

The present work shows that *A. mearnsii* and *A. retinodes* could be good sources of phenolic compounds, with extracts of the two species presenting significantly different compositions.

It is possible to conclude that gallic acid, syringaldehyde, and caffeic acid appear in the same quantity in *A. retinodes* and *A. mearnsii* in both flowering stages. Some compounds, namely furfural, 4-Hydroxybenzoic acid, chlorogenic acid, 4-hydroxybenzaldehyde, myricitrin, rutin, kaempferol, and myricetin, appear only in *A. retinodes* while (+)-catechin and coniferaldehyde were only quantifiable in *A. mearnsii* flowers. For the compounds present only in *A. retinodes*, almost all presented statistical differences between flowering stages. Compounds common to both species, such as vanillin, *p*-coumaric acid, trans-cinnamic acid, 5-methyfurfural, quercetin, 4′,5,7-trihydroxyflavanone, catechol, and (-)-epicatechin, were generically found in higher concentrations in *A. mearnsii* flowers. The compounds 5-methyfurfural, quercetin, catechol, and (-)-epicatechin showed significant variability regarding flowering stage. The phenolic compounds present in the Acacia flowers might be interesting, with the potential to harvest them at preliminary flowering stages being another tool to improve the control and dissemination of these invasive alien species.

It was possible to identify the potentiality of FTIR-ATR to distinguish between the different flower extracts, given their chemical composition.

The information regarding the flowers of these species is scarce, so we believe that more studies are needed to assess their potential application and value-added products.

## Figures and Tables

**Table 1:** Total phenolic compounds and flavonoids content (TPC and TFC) (mean ± standard deviation) of *A. retinodes* and *A. mearnsii* flowers extracts with different flowering stages.

	FS	TPC (mg GAE/g Extract)	TFC (mg QE/g Extract)
***A. retinodes***	EF	311.24 ± 23.36 b	136.47 ± 1.27 a
	LF	350.50 ± 13.79 b	287.91 ± 14.28 b
***A. mearnsii***	EF	310.55 ± 12.76 b	317.97 ± 1.89 c
	LF	300.03 ± 2.36 a	342.73 ± 4.32 d
Species (S)		49.1 ***	46.8 ***
Flowering stages (FS)		n.s.	25.9 ***
SxFS		n.s.	26.6 ***
Residual		50.9	0.7

FS—flowering stages; EF—early flower; LF—late flower; TPC – total phenolic compounds; TFC—flavonoids content; n.s. for *p* > 0.05, *** *p* < 0.001; GAE—gallic acid equivalents; QE—quercetin equivalents. Means within the same column followed by different letters are significantly different (*p* < 0.05) according to the LSD Test.

**Table 2:** Detected compounds in *A. retinodes* and *A. mearnsii* flowers by UHPLC/ESI-QTOF-MS.

Compound	t_R_ (min)	m/z Experimental
**Polyphenols Analysed in Negative Ionization Mode**		
**Phenolic acids**		
**Hydroxybenzoic acids**		
Gallic acid	3.0	169.0142
Protocatechuic acid	6.4	153.0193
4-Hydroxybenzoic acid	8.3	137.0244
Gentisic acid	8.3	153.0193
Salicylic acid	11.5	137.0244
**Hydroxy cinnamic acids**		
5-*O*-Chlorogenic acid	8.9	353.0878
Caffeic acid	9.3	179.0350
*p*-Coumaric acid	10.4	163.0400
**Hydroxycoumarins**		
Aesculetin	9.1	177.0193
**Flavonoids**		
**Dihydrochalcones**		
Phlorizin	11.3	435.1296
**Flavanones**		
Naringenin (aglycone)	12.5	271.0612
**Flavones**		
Chrysin (aglycone)	15.1	253.0506
**Flavonols**		
(+)-Catechin	8.5	289.0717
Quercetin-3-*O*-rhaminoside	9.2	447.0933
(-)-Epicatechin	9.5	289.0717
Quercetin-3-*O*-galactoside	11.0	463.0882
Quercetin-3-*O*-glucoside	11.0	463.0882
Quercetin-3-*O*-rutinoside	11.0	609.1461
Kaempferol 3-*O*-glucoside	11.5	447.0933
Kaempferol 3-*O*-rutinoside	11.5	593.1512
Quercetin (aglycone)	12.0	301.0354
Luteolin (aglycone)	12.7	285.0404
Kaempferol (aglycone)	13.3	285.0404
**Other polyphenols**		
Catechol	6.8	109.0295
**Polyphenols analysed in positive ionization mode**		
**Anthocyanines**		
Delphinidin 3-*O*-rutinoside *	9.1	610.1539
Cyanidin-3-*O*-glucoside	9.4	449.1084
Delphinidin (aglycone)	11.1	304.0578
Peonidin 3-*O*-glucoside	18.4	463.1240

* analyzed in ESI (-) mode; **t_R_**—retention time.

**Table 3:** The concentration of phenolic compounds (µg/g) in the ethanol extracts from *A. retinodes* and *A. mearnsii* flowers by HPLC-DAD using analytical standards and calibration curves (mean ± standard deviation).

Compound	t_R_ (min)	λ_max_ (nm)	*A. retinodes*		*A. mearnsii*	
			EF	LF	EF	LF
**Simple phenolics**						
Catechol	5.7	280	4.53 ± 0.23 c	6.20 ± 0.21 d	2.50 ± 0.08 a	3.64 ± 0.10 b
**Hydroxybenzoic acids**						
Gallic acid	3.1	280	1.45 ± 0.15 a	4.38 ± 1.27 a	5.88 ± 3.35 a	6.07 ± 1.06 a
4-Hydroxybenzoic acid	9.8	255	4.86 ± 0.63 a	7.51 ± 0.38 b	<LOQ ▪	<LOQ ▪
**Hydroxybenzoic aldehydes**						
4-Hydroxybenzaldehyde	12.6	280	4.80 ± 0.07 a	7.50 ± 0.42 b	<LOQ ▪▪	<LOQ ▪▪
Vanillin	16.6	280	7.55 ± 0.52 a	31.8 ± 4.24 a	194.93 ± 10.52 b	310.71 ± 65.68 c
Syringaldehyde	20.9	322	1.51 ± 0.55 ab	1.76 ± 0.70 ab	2.45 ± 0.83 b	0.36 ± 0.25 a
**Hydroxycinnamic acids**						
Chlorogenic acid	10.0	280	65.84 ± 3.15 b	12.16 ± 2.35 a	<LOQ ▪▪	<LOQ ▪▪
Caffeic acid	12.9	322	7.59 ± 1.16 b	5.84 ± 0.89 ab	6.92 ± 2.46 ab	3.40 ± 0.34 a
*p*-Coumaric acid	19.8	291	0.74 ± 0.02 a	1.45 ± 0.21 a	11.94 ± 2.30 b	10.35 ± 1.05 b
trans-Cinnamic acid	33.5	280	0.68 ± 0.06 a	1.66 ± 0.09 a	9.65 ± 3.30 b	16.17 ± 3.91 b
**Hydroxycinnamic aldehydes**						
Coniferaldehyde	29.2	322	0.44 ± 0.01 a	0.43 ± 0.05 a	<LOQ ▪▪	<LOQ ▪▪
**Furans**						
Furfural	5.7	2.63	0.48 ± 0.03 a	3.68 ± 0.99 b	<LOQ ▪▪▪	<LOQ ▪▪▪
5-Methylfurfural	11.6	255	42.99 ± 6.29 b	219.71 ± 20.14 c	1.71 ± 1.14 a	15.09 ± 2.01 ab
**Flavonoids**						
**Flavanols**						
(+)-Catechin	9.0	280	<LOQ ▪▪▪	<LOQ ▪▪▪	0.26 ± 0.12 a	0.15 ± 0.01 a
(-)-Epicatechin	10.6	280	9.43 ± 0.82 c	14.38 ± 0.70 d	3.05 ± 0.08 a	7.07 ± 0.43 b
**Flavonols**						
Rutin	31.9	255	0.70 ± 0.04 a	4.31 ± 0.72 b	<LOQ ▪▪	<LOQ ▪▪
Myricitrin	33.4	263	39.49 ± 3.54 a	70.39 ± 3.54 b	<LOQ ▪▪	<LOQ ▪▪
Myricetin	34.5	360	2.63 ± 0.10 a	8.71 ± 0.09 b	<LOQ ▪▪	<LOQ ▪▪
Quercetin	41.0	360	1.11 ± 0.02 a	5.54 ± 0.11 c	0.94 ± 0.08 a	2.78 ± 0.19 b
Kaempferol	45.3	360	7.62 ± 1.23 b	2.32 ± 1.32 a	<LOQ ▪▪	<LOQ ▪▪
**Flavones**						
4′,5,7-Trihydroxyflavanone	43.5	280	2.35 ± 0.20 a	1.53 ± 0.42 a	5.92 ± 0.98 b	5.59 ± 0.53 b

**t_R_**—retention time; λ_max_—maximum wavelength; EF—Early flower; LF—late flower; LOQ—Limit of quantification. LOQ ▪ (4-Hydroxybenzoic acid) 3.13 µg/mL; LOQ ▪▪ (4-Hydroxybenzaldehyde; caffeic acid; chlorogenic acid; coniferaldehyde; kaempferol; myricetin; quercetin; myricitrin; rutin) 0.78 µg/mL; LOQ ▪▪▪ (furfural; (+)-catechin) 1.56 µg/mL; Means within the same row followed by different letters are significantly different (*p* < 0.05) according to the LSD Test.

**Table 4:** Variance percentages were obtained in a two-way ANOVA performed for all analysed compounds concerning Acacia flowers from two species and two flowering stages.

Compound	Specie (S)	Flowering Stages (FS)	S × FS	Residual
**Appear in booth Acacia species**				
Gallic acid	n.s.	n.s.	n.s.	--
Vanillin	89.1 ***	7.2 *	n.s.	3.7
Syringaldehyde	n.s.	n.s.	n.s.	--
Caffeic acid	n.s.	n.s.	n.s.	--
*p*-Coumaric acid	96.9 ***	n.s.	n.s.	0.6
*trans*-Cinnamic acid	91.1 **	n.s.	n.s.	8.9
5-Methyfurfural	40.2 ***	24.0 ***	35.3 ***	0.6
Quercetin	14.0 ***	64.2 ***	21.6 ***	0.2
4′,5,7-Trihydroxyflavanone	95.2 ***	n.s.	n.s.	4.8
Catechol	71.9 ***	27.0 ***	1.0 ***	0.1
(-)-Epicatechin	69.8 ***	29.6 ***	0.3 ***	0.2
**Appear only in *A. retinodes***				
Furfural		90.9 *		9.1
4-Hydroxybenzoic acid		99.9 ***		0.1
Chlorogenic acid		99.5 **		0.5
4-Hydroxybenzaldehyde		97.5 *		2.5
Myricitrin		97.0 *		3.0
Rutin		100 ***		0.0
Kaempferol		n.s.		--
Myricetin		100 ***		0.0
**Appear only in *A. mearnsii***				
(+)-Catechin		n.s.		--
Coniferaldehyde		n.s.		--

n.s. for *p* > 0.05; * 0.01 < *p* < 0.05; ** 0.001 < *p* < 0.01; *** *p* < 0.001.

**Table 5:** Estimated concentration of phenolic compounds (µg/g) in the ethanol extracts from *A. retinodes* and *A. mearnsii* flowers by UHPLC/ESI-QTOF-MS (mean ± standard deviation).

Compound	*A. retinodes*		*A. mearnsii*	
	EF	LF	EF	LF
**Analysed in Negative Ionization Mode**				
Aesculetin	2.48 × 10^−2^ ± 3.36 × 10^−4^	0.014 ± 0.2 × 10^−2^	0.097 ± 0.2 × 10^−3^	0.014 ± 0.1 × 10^−3^
Chrysin (aglycone)	7.65 × 10^−5^ ± 8.06 × 10^−6^	1.15 × 10^−4^ ± 1.02 × 10^−5^	1.04 × 10^−4^ ± 1.61 × 10^−6^	1.28 × 10^−4^ ± 8.90 × 10^−7^
Delphinidin 3-*O*-rutinoside	1.25 ± 0.10	2.46 ± 0.05	0.42 ± 0.002	1.87 ± 0.03
Gentisic acid	0.31 ± 0.3 × 10^−2^	0.32 ± 0.1 × 10^−2^	0.50 ± 0.02	0.50 ± 0.02
kaempferol (aglycone)	11.18 ± 0.51	32.93 ± 0.34	2.40 ± 0.01	1.94 ± 0.09
kaempferol 3-*O*-glucoside	0.80 ± 0.02	1.47 ± 0.02	9.63 ± 0.48	9.43 ± 0.18
Luteolin (aglycone)	0.95 ± 0.02	1.19 ± 0.001	0.55 ± 0.02	0.52 ± 0.01
Naringenin (aglycone)	0.72 ± 0.02	0.63 ± 0.04	1.14 ± 0.02	1.16 ± 0.03
Nicotiflorin	0.03 ± 0.1 × 10^−2^	0.04 ± 0.3 × 10^−2^	0.01 ± 0.1 × 10^−3^	0.52 × 10^−2^ ± 0.7 × 10^−2^
Phlorizin	49.70 ± 4.65	56.09 ± 1.51	37.43 ± 16.28	40.98 ± 21.49
Protocatechuic acid	0.02 ± 0.1 × 10^−2^	0.45 ± 0.02	0.06 ± 0.21 × 10^−3^	0.04 ± 2.2 × 10^−3^
salicyclic acid	0.05 ± 0.2 × 10^−2^	0.08 ± 0.2 × 10^−2^	0.45 x 10^−2^± 0.1 × 10^−3^	0.04 ± 0.2 × 10^−2^
Quercetin (aglycone)	2.08 ± 0.32	11.59 ± 0.64	0.70 ± 0.003	1.55 ± 0.01
Quercetin-3-*O*-glucoside + Quercetin-3-*O*-galactoside (sum of isomers)	0.75 ± 0.02	4.21 ± 0.01	1.96 ± 0.01	3.59 ± 0.04
Quercetin-3-*O*-rhaminoside	0.79 ± 0.001	0.83 ± 0.11	17.60 ± 0.72	21.82 ± 0.62
**Analysed in positive ionization mode**				
Chrysanthemin	nd	nd	12.56 ± 1.36	14.42 ± 0.29
Peonidin 3-*O*-glucoside	0.37 ± 0.01	0.83 ± 0.002	0.49 ± 0.14	0.90 ± 0.09
Delphinidin	2.96 ± 0.02	11.46 ± 0.17	4.68 ± 0.01	9.44 ± 0.44

EF—Early flower; LF—late flower; nd—not detected.

**Table 6:** Mass spectrum data and peak assignments for the most abundant compounds identified from the *A.* *mearnsii* and *A. retinodes* extracts, with annotation level MS/MS.

*A. mearnsii* Extracts							
Monoisotopic Mass	Area	t_R_ (min)	[M−H]^−^	[M+H]^+^	Tentative Annotation		Annotation Level
			(*m/z*)	(*m/z*)			
**Hydroxycinnamic acid glycosides**							
326.2282	3.38 × 10^6^	9.1	325.0935	327.1358		*p*-coumaroyl hexose	MS/MS
356.2498	2.28 × 10^6^	9.3	355.104	357.1155		feruloyl hexose	MS/MS
**Flavanones *O*-glycosides**							
434.3828	3.26 × 10^7^	10.2/10.9/11.5/11.9	433.115	435.0927		naringenin *O*-hexose isomers	MS/MS
**Flavonol *O*-glycosides**							
448.2944	3.11 × 10^6^	11.6	447.0943	449.1090		quercetin *O*-hexoside	MS1
*A. retinodes* extracts							
**Monoisotopic mass**	**Area**	**t_R_ (min)**	**[M−H]^−^**	**[M+H]^+^**		**Tentative annotation**	**Annotation level**
			**(*m/z*)**	**(*m/z*)**			
**Hydroxycinnamic acid glycosides**							
326.2282	3.38 × 10^6^	9.1	325.0935	327.1358		*p*-coumaroyl hexose	MS/MS
356.2498	2.28 × 10^6^	9.3	355.104	357.1155		feruloyl hexose	MS/MS
**Flavanones *O*-glycosides**							
434.3828	4.94 × 10^6^	11.1/11.3/11.5	433.115	435.0927		naringenin *O*-hexose isomers	MS/MS
**Flavonol *O*-glycosides**							
788.2021	9.94 × 10^5^	9.7	787.1949	789.2089		quercetin *O*-triglucoside	MS1
642.1445	1.27 × 10^6^	9.8	641.1372	643.1517		myricetin *O*-dihexoside isomer	MS1
626.1483	3.92 × 10^6^	10.1/10.3	625.1423	627.1571		quercetin *O*-dihexoside isomer	MS1
480.0904	2.29 × 10^6^	10.5	479.0839	481.0982		myricetin *O*-hexoside isomer	MS1
450.0798	2.50 × 10^6^	10.8	449.0703	451.0879		myricetin *O*-hexoside isomer	MS1
464.0962	2.05 × 10^6^	10.9/11.1	463.0861	465.1034		quercetin *O*-hexoside isomer	MS1
448.2944	3.11 × 10^6^	11.6	447.0943	449.1090		quercetin *O*-hexoside isomer	MS1
318.0378	6.74 × 10^6^	12.4	317.0305	319.0454		myricetin (aglycone)	MS/MS
302.0431	1.47 × 10^6^	12.5	301.0358	303.0502		quercetin (aglycone)	MS/MS
**Jasmonic acids**							
388.1742	1.39 × 10^6^	9.8	387.17	389.18		11-hydroxyjasmonic acid glucoside	MS1

## Data Availability

Not applicable.

## Supplementary Materials

The following supporting information can be downloaded at: https://www.mdpi.com/article/10.3390/plants11111442/s1, Table S1. Data validation (analysis by HPLC/DAD). Table S2. Data validation (analysis by UHPLC/ESI-QTOF-MS). Figure S1. Representative Base Peak Chromatogram (BPC) profile (negative and positive ion mode) from the extract of *A. maernsii*. The *m/z* values for the most abundant compounds are given. Figure S2. Representative Base Peak Chromatogram (BPC) profile (negative and positive ion mode) from the extract of *A. retinodes*. The *m/z* values for the most abundant compounds are given.
