# Supplementary material for: Characterisation of the Phenolic Profile of Acacia retinodes and Acacia mearnsii Flowers’ Extracts
Source: Plants (Basel). 2022 May 28;11(11):1442. doi: 10.3390/plants11111442 (PMC9182983; doi:10.3390/plants11111442)
Supplement: Supplementary file 1 [file plants-11-01442-s001.zip › plants-1732809-Supplementary.pdf]

Supplementary Material

Table S1. Data validation (analysis by HPLC/DAD)

Compound	Linear range (μg/mL)	Linearity*			
		Slope	Intercept	R^{2*}	Lower Limit of quantitation (μg/mL)
Catechol	3.13 - 100	39.35 ± 5.51	-33.47 ± 2.71	0.9989 ± 0.0015	3.13
Gallic acid	0.78 - 50	175.90 ± 14.95	-12.66 ± 1.30	0.9999 ± 0.0002	0.78
4-Hydroxybenzoic acid	3.13 - 100	412.17 ± 20.20	567.21 ± 83.95	0.9976 ± 0.0018	3.13
4-Hydroxybenzaldehyde	0.78 - 50	524.51 ± 52.45	-73.08 ± 3.07	0.9999 ± 0.0009	0.78
Vanillin	0.78 - 25	97.82 ± 14.28	-31.23 ± 3.19	0.9998 ± 0.0012	0.78
Syringaldehyde	1.56 - 50	239.73 ± 5.75	150.48 ± 21.82	0.9989 ± 0.0047	1.56
Chlorogenic acid	0.78 - 50	39.23 ± 2.24	-39.50 ± 2.33	0.9986 ± 0.0033	0.78
Caffeic acid	0.78 - 50	125.95 ± 11.08	-53.10 ± 1.49	0.9971 ± 0.0025	0.78
p-Coumaric acid	0.78 - 50	311.57 ± 44.87	-195.51 ± 15.05	0.9998 ± 0.0015	0.78
trans-Cinnamic acid	0.78 - 25	1531.00 ± 104.11	-530.96 ± 60.53	0.9999 ± 0.0008	0.78
Coniferaldehyde	0.78 - 50	150.70 ± 22.45	-74.19 ± 7.12	0.9996 ± 0.0011	0.78
Furfural	1.56 - 50	907.15 ± 17.24	-434.15 ± 46.15	0.9998 ± 0.0004	1.56
5-Methylfurfural	1.56 - 50	177.18 ± 9.57	-125.51 ± 9.04	0.9998 ± 0.0017	1.56
(+)-Catechin	1.56 - 50	23.78 ± 2.81	-10.05 ± 0.55	0.9993 ± 0.0006	1.56
(-)-Epicatechin	1.56 - 50	16.97 ± 2.55	-6.77 ± 0.29	0.9997 ± 0.0027	1.56
Rutin	0.78 - 50	31.52 ± 3.22	-38.66 ± 3.29	0.9995 ± 0.0014	0.78
Myricitrin	0.78 - 50	27.87 ± 2.56	-12.10 ± 1.71	0.9998 ± 0.0008	0.78
Myricetin	0.78 - 50	51.36 ± 3.13	-9.42 ± 1.42	0.9999 ± 0.0010	0.78
Quercetin	0.78 - 50	51.50 ± 7.57	-2.47 ± 0.24	0.9998 ± 0.0005	0.78
Kaempferol	0.78 - 50	58.83 ± 6.59	6.75 ± 0.83	0.9998 ± 0.0003	0.78
4',5,7-Trihydroxyflavanone	0.78 - 25	239.15 ± 11.24	-48.62 ± 7.39	0.9998 ± 0.0009	0.78

Table S2. Data validation (analysis by UHPLC/ESI-QTOF-MS)

Polyphenols analysed in negative ionization mode					
**Compounds**	**Linear range (µg/mL)**	**Linearity**			
		**Slope**	**Intercept**	**R^2**	**Lower limit of quantitation (µg/mL)**
Gentisic acid	0.125- 5	4.84E+05	7.59E+04	0.997	0.125
Protocatechuic acid	0.5-5	8.16E+04	5.52E+03	0.998	0.5
Salicylic acid	0.125- 5	7.62E+05	3.30E+05	0.990	0.125
Aesculetin	0.125- 5	2.95E+06	9.22E+05	0.990	0.125
Phlorizin	0.125- 5	6.80E+05	2.29E+05	0.984	0.125
Naringenin (aglycone)	Response factor:	2.67E+05			
Chrysin (aglycone)	0.5-10	3.56E+07	9.90E+07	0.990	0.5
Kaempferol (aglycone)	0.5-10	1.47E+05	9.44E+04	0.997	0.5
Kaempferol 3-*O*-glucoside	0.125- 5	9.23E+05	1.00E+05	0.997	0.125
Kaempferol 3-*O*-rutinoside	0.125- 5	7.34E+05	2.02E+05	0.990	0.125
Luteolin (aglycone)	0.125- 5	4.07E+05	6.16E+04	0.996	0.125
Quercetin (aglycone)	0.125- 5	9.44E+05	6.04E+04	0.996	0.125
Quercetin-3-*O*-galactoside	0.5-5	2.58E+05	2.74E+04	0.999	0.5
Quercetin-3-*O*-glucoside	0.125- 5	9.03E+05	4.16E+04	0.999	0.125
Quercetin-3-*O*-rhaminoside	0.125- 5	6.36E+05	1.69E+05	0.990	0.125
Delphinidin 3-*O*-rutinoside*	Response factor:	9.69E+03			
**Polyphenols analysed in positive ionization mode**					
**Compounds**	**Linear range (µg/mL)**	**Linearity**			
		**Slope**	**Intercept**	**R^2**	**Lower limit of quantitation (µg/mL)**
Cyanidin-3-*O*-glucoside	0.5-10	1.44E+06	7.75E+05	0.992	0.5
Peonidin 3-*O*-glucoside	Response factor:	5.93E+05			
Delphinidin (aglycone)	Response factor:	1.33E+04			

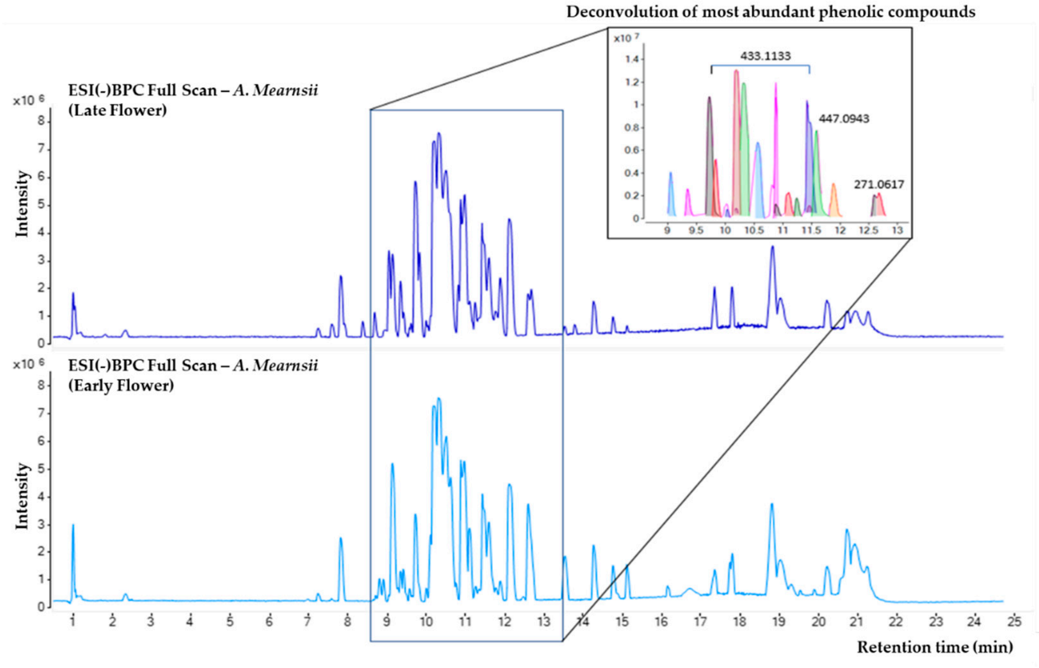

Figure S1. Representative Base Peak Chromatogram (BPC) profile (negative and positive ion mode) from the extract of *A. maernsii*. The *m*/*z* values for the most abundant compounds are given.

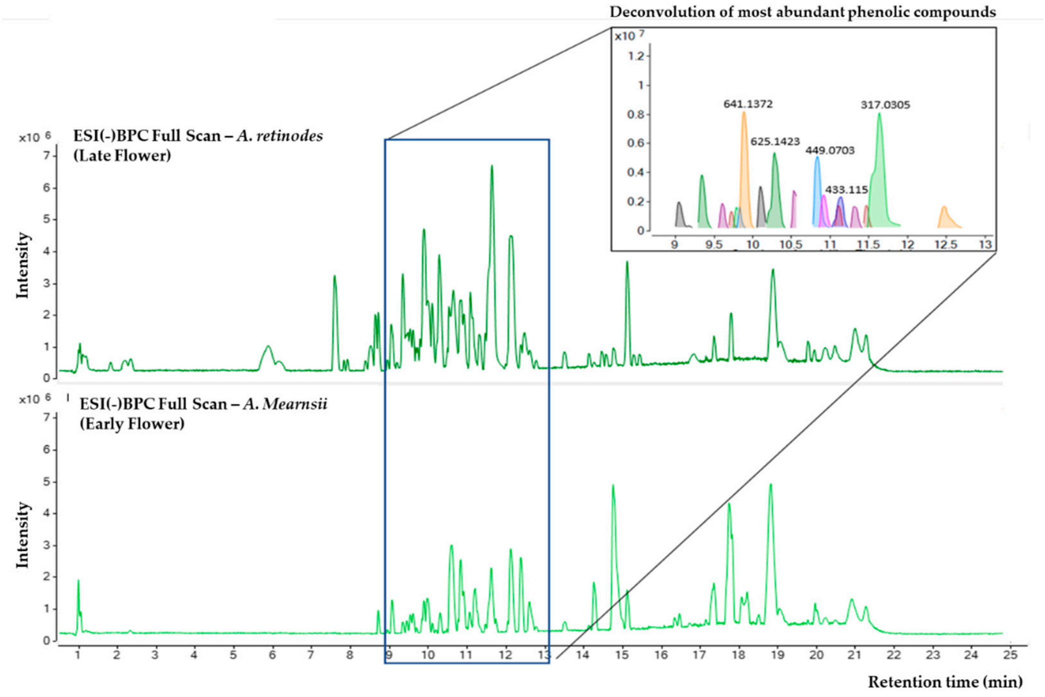

Figure S2. Representative Base Peak Chromatogram (BPC) profile (negative and positive ion mode) from the extract of *A. retinodes*. The *m*/*z* values for the most abundant compounds are given.
